# Correction: Qiu et al. P53 Deficiency Accelerates Esophageal Epithelium Intestinal Metaplasia Malignancy. *Biomedicines* 2023, *11*, 882

**DOI:** 10.3390/biomedicines13081978

**Published:** 2025-08-14

**Authors:** Quanpeng Qiu, Gang Guo, Xiaolong Guo, Xiake Hu, Tianyu Yu, Gaixia Liu, Haowei Zhang, Yinnan Chen, Junjun She

**Affiliations:** 1 Department of General Surgery, The First Affiliated Hospital of Xi’an Jiaotong University, Xi’an 710061, China; 2 Center for Gut Microbiome Research, Med-X Institute, The First Affiliated Hospital of Xi’an Jiao Tong University, Xi’an 710061, China; 3 Department of High Talent, The First Affiliated Hospital of Xi’an Jiaotong University, Xi’an 710061, China

## Error in Figure

In the original publication [1], there was a mistake in Figure 6E, as published. We have identified that Figure 6E (OE19-Ctrl) in the manuscript was inadvertently duplicated from Figure 6D (OE33-Ctrl) due to an oversight during figure preparation, and we propose to correct it as follows. The corrected Figure 6E (OE19-Ctrl) appears below. The authors state that the scientific conclusions are unaffected. This correction was approved by the Academic Editor. The original publication has also been updated.

## Title Typographical Error

Additionally, a typographical error in the article title has been corrected, changing “Accelerate” to “Accelerates”.

## Figures and Tables

**Figure 6:**
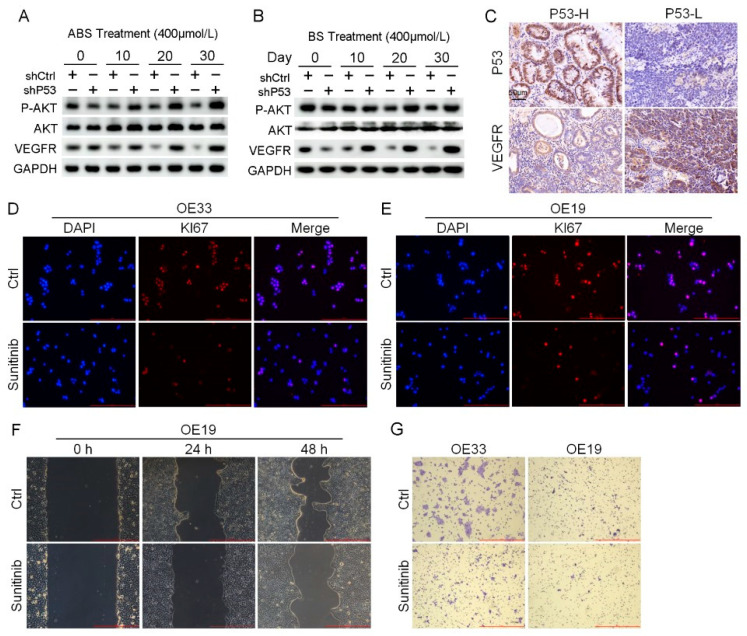
Venerability of *VEGFR* signaling pathway in *P53* deficient EAC cells. (**A**) The protein expression level of *AKT*, *VEGFR* and phosphorylation of *AKT*. (**B**) The protein expression level of *AKT*, *VEGFR* and phosphorylation of *AKT*. (**C**) Representative images of *P53* and *VEGFR* IHC staining. (**D**,**E**) Representative images of OE33 cells (**D**) and OE19 cells (**E**) by *Ki67* immunofluorescence after treatment with 2 μmol/L Sunitinib. (**F**) OE33 cell migration capacity after treatment with 2 μmol/L Sunitinib. (**G**) Transwell assay for OE33 and OE19 cell lines after treatment with 2 μmol/L Sunitinib. +: Treated accordingly; −: Not treated accordingly. Data were statistically analyzed using the student *t*-test.
