# Supplementary material for: P53 Deficiency Accelerates Esophageal Epithelium Intestinal Metaplasia Malignancy
Source: Biomedicines. 2023 Mar 13;11(3):882. doi: 10.3390/biomedicines11030882 (PMC10046085; doi:10.3390/biomedicines11030882)
Supplement: Supplementary file 1 [file biomedicines-11-00882-s001.zip › biomedicines-2236333-supplementary.pdf]

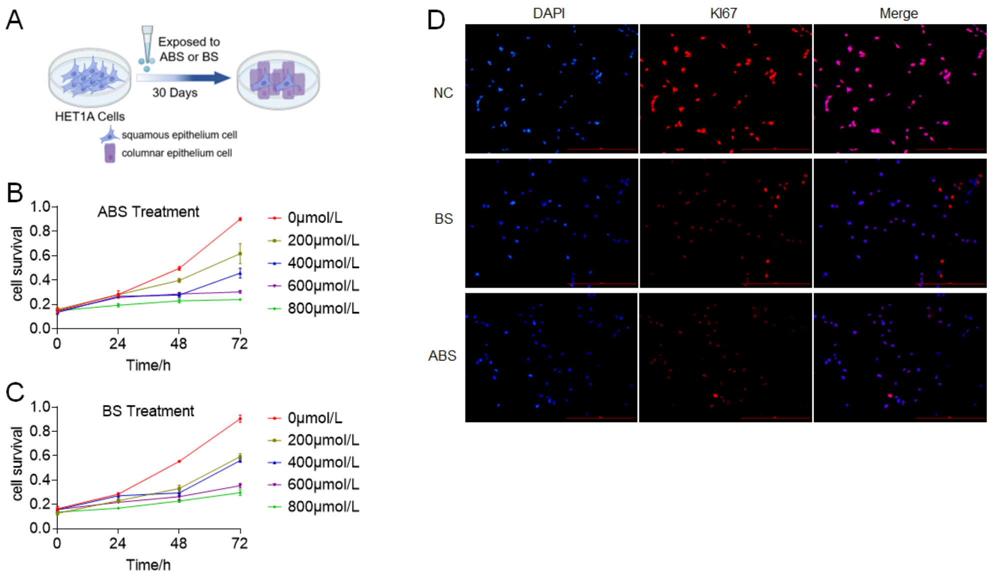

Figure S1. Dose-dependent inhibition of HET1A proliferation by long-term treatment of ABS or BS. (**A**) Schematic outline of HET1A cells continuous exposure to ABS and BS. (**B**) Proliferation of HET1A cells exposure to ABS with different concentration. (**C**) Proliferation of HET1A cells exposure to BS with different concentration. (**D**) Representative immuno-fluorescence of KI67 images of HET1A cells with 400 μmol/L ABS or BS treatment for 48 hours.

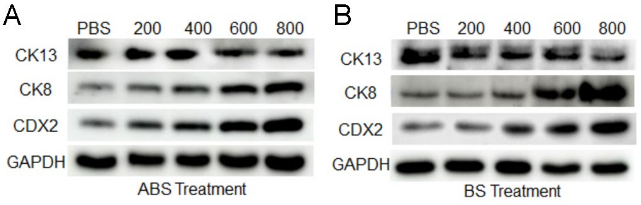

Figure S2. Bile acids promote the intestinal epithelial metaplasia of HET1A. (**A**) CK13, CK8 and CDX2 protein expression after treatment with ABS titration in HET1A cells after 72 hours. (**B**) CK13, CK8 and CDX2 protein expression after treatment with BS titration in HET1A cells after 72 hours.

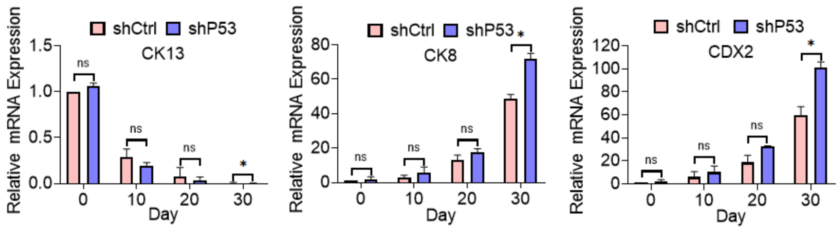

Figure S3. P53 deficiency affects bile acid-mediated intestinal metaplasia. CK13, CK8 and CDX2 mRNA expression after treatment with ABS. Data were statistically analyzed using Student t test. * $p < 0.05$, ns: none significance.

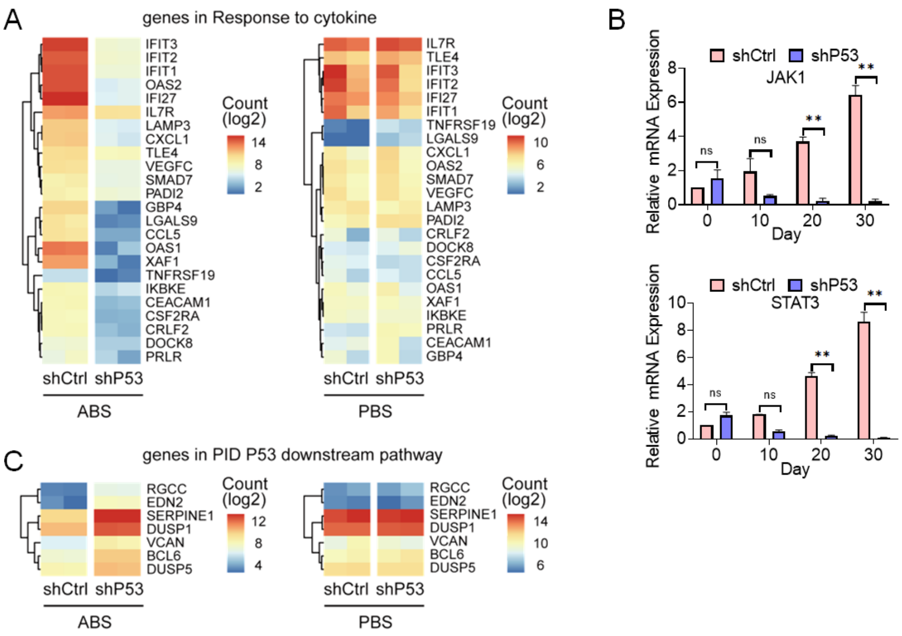

Figure S4. Enriched pathways for P53-dependent differentially expressed genes after ABS treatment. (**A**) Heatmap of gene expression in response to cytokine pathway after exposure to ABS (left) and PBS (right) of P53 knockdown and control group. (**B**) JAK1 and STAT3 mRNA expression after treatment with ABS. (**C**) Heatmap of gene expression in PID P53 downstream pathway after exposure to ABS (left) and PBS (right) of P53 knockdown and control group. Data were statistically analyzed using Student t test. ** $p < 0.01$, ns: none significance.
